# A scoping review of academic and grey literature on migrant health research conducted in Scotland

**DOI:** 10.1186/s12889-024-18628-1

**Published:** 2024-04-25

**Authors:** G. Petrie, K. Angus, R. O’Donnell

**Affiliations:** 1 Caledonia House, NHS Greater Glasgow and Clyde, Glasgow, Scotland UK; 2 Institute for Social Marketing and Health, Faculty of Health Sciences and Sport, University of Stirling, Stirling, FK9 4LA Scotland UK

**Keywords:** Asylum seekers, Scoping review, Refugees, Research funding, Immigration

## Abstract

**Background:**

Migration to Scotland has increased since 2002 with an increase in European residents and participation in the Asylum dispersal scheme. Scotland has become more ethnically diverse, and 10% of the current population were born abroad. Migration and ethnicity are determinants of health, and information on the health status of migrants to Scotland and their access to and barriers to care facilitates the planning and delivery of equitable health services. This study aimed to scope existing peer-reviewed research and grey literature to identify gaps in evidence regarding the health of migrants in Scotland.

**Methods:**

A scoping review on the health of migrants in Scotland was carried out for dates January 2002 to March 2023, inclusive of peer-reviewed journals and grey literature. CINAHL/ Web of Science/SocIndex and Medline databases were systematically searched along with government and third-sector websites. The searches identified 2166 journal articles and 170 grey literature documents for screening. Included articles were categorised according to the World Health Organisation’s 2016 Strategy and Action Plan for Refugee and Migrant Health in the European region. This approach builds on a previously published literature review on Migrant Health in the Republic of Ireland.

**Results:**

Seventy-one peer reviewed journal articles and 29 grey literature documents were included in the review. 66% were carried out from 2013 onwards and the majority focused on asylum seekers or unspecified migrant groups. Most research identified was on the World Health Organisation’s strategic areas of right to health of refugees, social determinants of health and public health planning and strengthening health systems. There were fewer studies on the strategic areas of frameworks for collaborative action, preventing communicable disease, preventing non-communicable disease, health screening and assessment and improving health information and communication.

**Conclusion:**

While research on migrant health in Scotland has increased in recent years significant gaps remain. Future priorities should include studies of undocumented migrants, migrant workers, and additional research is required on the issue of improving health information and communication.

**Supplementary Information:**

The online version contains supplementary material available at 10.1186/s12889-024-18628-1.

## Background

 The term migrant is defined by the International Organisation for Migration as “*a person who moves away from his or her place of usual residence, whether within a country or across an international border, temporarily or permanently, and for a variety of reasons. The term includes several well-defined legal categories of people, including migrant workers; persons whose particular types of movements are legally-defined, such as smuggled migrants; as well as those whose status are not specifically defined under international law, such as international students.”* [1] Internationally there are an estimated 281 million migrants – 3.6% of the world population, including 26.4 million refugees and 4.1 million asylum seekers – the highest number ever recorded [2]. The UN Refugee Society defines the term refugee as “*someone who has been forced to flee his or her country because of persecution, war or violence…most likely, they cannot return home or are afraid to do so*.” The term asylum-seeker is defined as *“someone whose request for sanctuary has yet to be processed.”* [3].

Net-migration to Europe was negative in the 19th century due to higher levels of emigration, however in the mid-20th century immigration began to rise, because of an increase in migrant workers and following conflicts in the Middle East and North Africa [4]. Current migration drivers include conflicts alongside world-wide economic instability, exacerbated by the Covid-19 pandemic [5]. Environmental damage due to climate change is expected to inflate the number of asylum seekers entering Europe in future [6]. The increase in migration to Europe is not a short-term influx but a long-term phenomenon, and European nations must adapt and find solutions to resulting financial, safeguarding and health challenges [7]. 

Data on healthcare use by migrants in Europe is variable, which means cross-country comparisons are inadequate [8]. Many countries do not record migration information within health records and all use disparate criteria to classify migrant status. The lack of comparative data hinders public health surveillance and effective interventions [9]. Even where information is available, results can be contradictory due to the multifarious migrant population. Migrants have a wide range of origin countries, socio-economic position, age and journeys undertaken which can affect health status [10]. 

Migrants initially may have better health than the general population, known as the ‘Healthy Migrant effect’ [11]. However, health declines with increasing length of residence [12] and over time to levels comparable with the general population [13]. Second generation immigrants may have higher mortality than average [14]. The process of acculturation to the host country, with adoption of unhealthy lifestyle and behaviours, increases the risk for chronic disease [15]. In addition, inequalities in health of migrants compared to host populations has been confirmed by wide-ranging research [16]. 

Host countries may limit healthcare access, with undocumented migrants sometimes only entitled to emergency care [17]. Even when access is granted, inequitable services can affect quality of care due to language barriers and cultural factors [18]. Poor working/living conditions and discrimination can exacerbate health inequalities [12]. Processing facilities for asylum seekers are frequently overpopulated, stressful environments [19] and threat of deportation, lack of citizenship rights and integration can negatively affect health and access to care [20]. Undocumented workers are unprotected by health and safety legislation leading to dangerous working conditions and injuries [15]. 

A systematic review of migrant health in the European Union (EU) found migrants have worse self-perceived health than the general population [21]. Research evidence indicates increased prevalence of cardiovascular disease, diabetes, mental health disorders and adverse pregnancy outcomes. Exposure to conflict, harsh travel conditions and suboptimal vaccine programmes can mean higher risk of communicable disease [22]. Scoping reviews have also been conducted to describe trends within migration health research in the United Kingdom (UK) [23] and identify gaps for future research agendas in the UK [23] and in the Republic of Ireland [24]. 

Almost three-quarters (73%) of published migration health research in the UK has been conducted in England, focusing primarily on infectious diseases and mental health. There is limited evidence on the social determinants of health, access to and use of healthcare and structural and behavioural factors behaviours that influence migrant health in the UK [23]. By contrast, a large amount of the migration research conducted in the Republic of Ireland has focused on the social determinants of health, and on health system adaptations, with a paucity of research focusing on improving health information systems [24]. 

### Migration and Health in Scotland

Immigration to Scotland began to rise in 2003 with the expansion of the EU [25]. The population in Scotland increased from 5.11 million to 5.47 million between 2005 and 2020 and is predicted to continue rising until 2028 [26] despite low birth rates, with the increased population resulting from inward migration [27]. Scotland’s population is becoming more ethnically diverse [28] and susceptibility to different health conditions varies by ethnic group, which has implications for the planning and provision of health services [29]. 7% of the current Scottish population are non-UK nationals and 10% were born outside Britain. The commonest countries of origin were Poland, Ireland, Italy, Nigeria and India [30]. 

Within Scotland, linking health data to ethnicity is standard in order to monitor and improve health of minority groups [31]. Ethnic background can differ from country of birth which means migration status cannot be assumed [32], although health inequalities experienced by migrants often extend to affect all ethnic minority groups [33]. The Scottish Health and Ethnicity Linkage Study (SHELS) linked census data to health records of 91% of the population which has provided information on mortality and morbidity by ethnic group and country of birth [34]. SHELS research indicates that the white-Scottish population have a higher mortality rate than other ethnic groups. This may be consequent to the comparatively poor health of the Scottish population relative to other European nations: high mortality rates in the general population may cause a perception that the health of minorities is more advantageous than in reality [35]. 

Cezard et al’s [13] analysis of self-perceived health among people in Scotland found that being born abroad had a positive impact on health status. Health declined with increased length of residence, which may be explained by cultural convergence with the majority population. Allik et al. [36] compared health inequalities by ethnic background and found that with increasing age, health differences reduced thus people aged over 75 of all ethnicities had similar or worse health status than White-Scottish people. While working-age migrants appear to be healthier than the White Scottish population, it cannot be assumed that in future this would extend to older age groups.

Research has shown deprivation as a cause of heath inequalities among ethnic minority and migrant groups [37]. The socio-economic status of minority ethnic groups in Scotland is unusual, as most are of similar or higher status than the white-Scottish population [38]. Therefore, public health interventions targeting deprivation may not address risk-factors for ethnic minorities and migrants [36]. Further research on determinants of health in migrants can help with planning and design of inclusive policies.

The 2011 census indicated that 50% of immigrants lived in the cities of Edinburgh, Glasgow, and Aberdeen. Glasgow had a greater percentage of non-European immigrants due to participation in the Asylum dispersal programme [39]. 10% of UK asylum seekers are placed in Glasgow, but records are not kept following approval of asylum claims, therefore the size of the refugee population is unknown [40]. While immigration is controlled by the British government, in policy areas devolved to the Scottish government, refugees and asylum seekers have more rights than elsewhere in UK, including access to primary healthcare for undocumented migrants [40]. Despite the mitigating effect of Scottish policies, asylum seekers’ health is worsened by the asylum process and associated poverty, marginalisation, and discrimination [40]. Health deteriorates with increasing length of time in the asylum system [40] and asylum seekers and refugees have additional health needs and require enhanced support [41]. Research on the health needs of asylum seekers in Scotland is required to ensure adequate healthcare.

### Aim and objectives

While scoping reviews on migrant health have been carried out in Europe [12], Ireland [24] and the UK [23] none are currently specific to the Scottish context. Given the devolved government of Scotland and demographics described above, a targeted review would help to clarify research priorities, with the aim of improving health and health care within the migrant community in Scotland. This work therefore builds on the published scoping review of migrant health in the Republic of Ireland [24]. The authors recommend replication of the study in other countries to facilitate cross-country comparison. Our aim was to scope peer-reviewed research and grey literature on migrant health conducted in Scotland and identify any gaps in the evidence. Our objectives were to: [1] understand the extent of the available research by topic area [2] summarise the types of research already conducted, populations studied, topics covered and approaches taken [3], map the existing research conducted in Scotland and [4] identify areas for future research based on any gaps in the evidence identified.

## Methods

A scoping review was conducted as they can aid detection of evidence gaps [42] and allow incorporation of grey literature in topics with insufficient published research [43]. Arksey and O’Malley’s [44] five stage scoping review framework was used.

### Stage 1: identifying the research question

Arskey and O’Malley [44] suggest maintaining a broad approach to identifying the research question, in order to generate breadth of coverage. On this basis, and in line with the research question identified in the Villarroel et al. [24] scoping review, our research question was framed as follows: *What is the scope, main topics and gaps in evidence in the existing literature on health of international migrants living in Scotland?* Arksey and O’Malley [44] highlight the importance of defining terminology at the outset of scoping reviews. For consistency, we used the broad definition of ‘migrant’ as per Villaroel et al. [24], from the International Organisation for Migration (IOM) [1]. References to refugees or asylum seekers followed the United Nations Refugee Agency definitions [3].

### Stage 2: identifying relevant studies

Electronic database searches identified reports alongside a grey literature search, in line with Arskey and O’Malley’s [44] guidance to search for evidence via different sources. CINAHL, Web of Science, SocIndex and Medline academic databases were selected with input from co-authors. Search terms for the review were based upon those used by Villaroel et al. [24] with additional relevant terms from Hannigan et al. [9] The strategy combined three sets of terms for: Migrants (e.g., refugee, migrant, immigrant or newcomer), Scotland and Health. Both free text terms and index terms were used and adapted to the 4 academic databases and searches were run on 10th March 2023 (see Additional File 1 for database search strategies). Thirteen Government, University, and third-sector websites in Scotland were scoped for selection then hand-searched for grey literature (listed in Additional File 1).

### Stage 3: study selection

Net-migration to Scotland increased in the 2000s [27] hence a date range of January 2002-March 2023 was used to identify evidence. The search was limited to English only. Inclusion/exclusion criteria for the studies were based on those used by Villaroel et al. [24] and expanded upon following discussion with co-authors (see Table 1). Reports were included if based on primary or secondary research on the health of international migrants in Scotland and used qualitative, quantitative or mixed methods research design. International or UK based reports were only included if Scottish results were documented separately. Reports on the health of ethnic minority groups in Scotland was included if place of birth was recorded. Research on internal (non-international) migrants within Scotland, either moving from one Scottish area to another or from another part of the United Kingdom to Scotland, were excluded.

**Table 1:** Inclusion and exclusion criteria modified from Villaroel et al. [24]

Inclusion criteria	Articles were included if: • Research was based on primary or secondary data on the health of migrants in Scotland • Research was based on primary or secondary data on the health of migrants in the United Kingdom or Europe, including Scotland, provided that Scottish data was recorded separately within results • Research was based on primary or secondary data about the health of Minority Ethnic people in Scotland provided that place of birth was clearly recorded and effect of birthplace on results shown. • In English language • Published from January 2002 to March 2023 • Research incorporated qualitative, quantitative or mixed methods
Exclusion criteria	Articles were excluded if: • No analysis of primary or secondary data was included, i.e., editorial, discussion articles, study protocols, methods papers, literature reviews. • Medical case reports • Non-human studies • Non-migrant health studies, including studies on Minority Ethnic people who were born in Scotland. • Research on migrants from elsewhere in the United Kingdom • Migrant studies in other countries • Studies on internal migrants within Scotland either moving from one Scottish area to another or from another part of the United Kingdom to Scotland. • No full text available • Studies about migrants in Scotland, but not related to their health status and not conducted in healthcare settings

### Stage 4: data charting

All records were saved to RefWorks for screening. Records were first screened at title/abstract stage with 10% independently checked by the co-authors. The remaining reports were single screened using full text by the first author. Data from the included records was extracted and organised in tabular form under the following headings, which were agreed by team members: article type (peer-reviewed article or grey literature), publication date, geographical setting, study/intervention’s target population, funding, primary research focus on migrant health (y/n), study objective, data collection method, study design (qualitative/quantitative/mixed) and main finding. Reports were not critically appraised in this scoping review.

### Stage 5: collating, summarising and reporting results

A report (either a peer-reviewed journal article or grey literature report) is used as our unit of analysis. In order to present the range of research identified, reports were grouped by the different headings in our data charting table and the outcomes considered for relevance to our scoping review’s aim. Our Results summarise the recency, focus, study designs and funding sources of the identified research, followed by the geographical settings and whether Scotland was included in international research reports. Reports were grouped by their study population and further sub-divided by publication type and geographical area for summarising. Finally, the WHO’s European strategy and action plan (SAAP) for refugee and migrant health [7] is a policy framework designed to help governments and other stakeholders monitor and improve migrant health in Europe. There are nine strategic areas in the WHO’s SAAP, which prioritise the most salient issues. In line with Villaroel et al’s [24] approach and in order to compare scoping review outcomes, these areas were used to categorise the findings of this review. Each report was matched to the most appropriate SAAP:

1. Establishing a Framework for Collaborative Action.
2. Advocating for the right to health of refugees.
3. Addressing the social determinants of health.
4. Achieving public health preparedness and ensuring an effective response.
5. Strengthening health systems and their resilience.
6. Preventing communicable disease.
7. Preventing and reducing the risks caused by non-communicable disease.
8. Ensuring ethical and effective health screening and assessment.
9. Improving health information and communication.

The primary focus (aims and objectives) of each report was used to identify the relevant SAAP area/areas. To improve reliability, results were compared using coding criteria used in Villaroel et al’s study (MacFarlane 2023, personal communication, 31st May). 10% of the reports were checked by one co-author to ensure consistent coding to SAAP categories. Any instances of uncertainty in mapping reports to the relevant SAAP area/areas were discussed and resolved by team members.

## Results

**Fig. 1:**
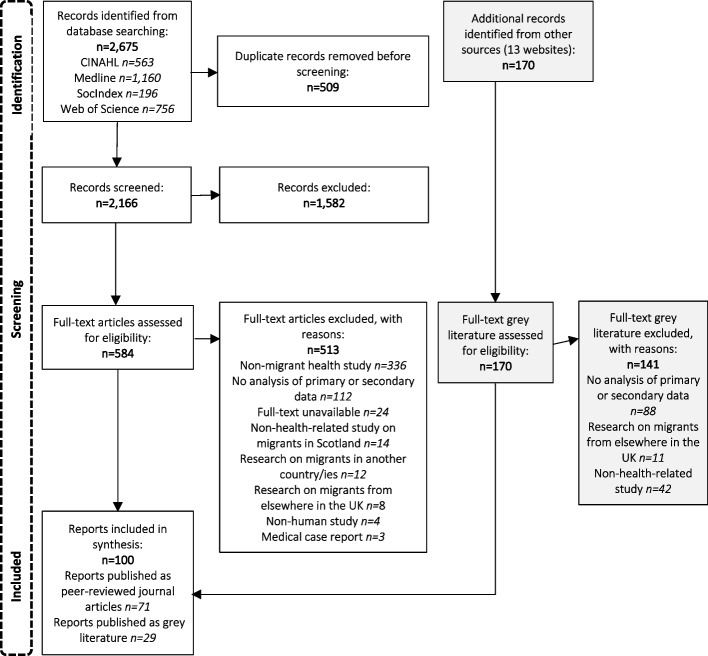
Flow chart illustrating the identification of sources of evidence included in the scoping review

This scoping review of the literature on migrant health in Scotland identified 2166 records from academic literature databases, following duplicate removal, and 170 records from website searches (see Fig. 1). Following screening, a total of 71 peer-reviewed journal articles and 29 grey literature studies (totalling 100 reports) were included for analysis (Results table and reference list are presented in Additional File 2).

### Overall findings

The majority of reports were published between 2013 and 2022. Fifty-eight reports (58%) focused exclusively on migrant health [18, 39, 45–102]. 23 centred on health but included other populations in addition to migrants – for example research on ethnic minorities or other vulnerable groups [13, 31, 35, 103–122]. Seventeen reports were included where the sample population were migrants, but the primary topic was not health – for example destitution, integration, and service needs [27, 73, 74, 123–135]. Health data was reported as part of the wider subject matter. One report [136] looked at the social determinants of breastfeeding including migrant status and one [137] compared attitudes to aging and family support between countries.

**Fig. 2:**
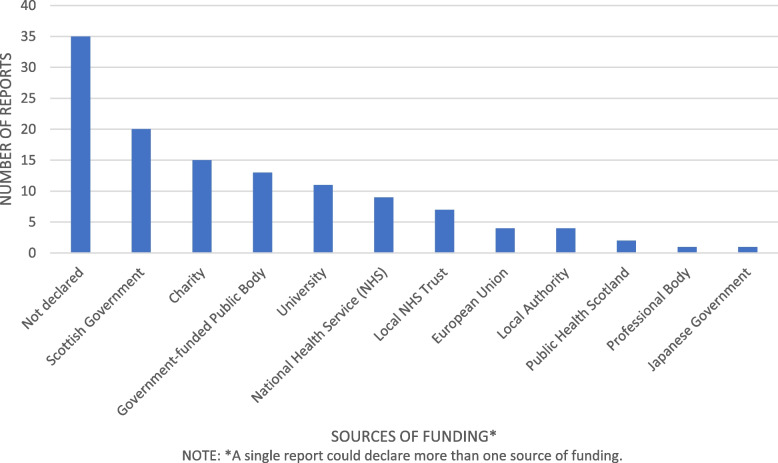
Sources of funding for migrant health research in Scotland

Funding sources were not declared for 35 (35%) of reports. The Scottish Government funded 20 reports (20%) [13, 27, 32, 39, 45–47, 66, 77, 88, 99–102, 113, 116, 119, 121, 129, 134]. Other common sources of funding included Government funded public bodies (*n* = 13) [45, 48–53, 104, 107, 113, 116, 131, 136], the Scottish Health Service (*n* = 18) (either the National Health Service (NHS) [13, 54, 56–59, 102, 113, 116], local NHS trusts [45, 60, 61, 77, 102, 103, 112] or by Public Health Scotland [13, 113]) Eleven reports (11%) were funded by Universities. The charity sector financed 15 (15%) reports [53, 63, 66, 69–74, 103, 111, 123, 125, 132, 138] and the EU and Scottish local authorities funded four reports each [45, 62, 75–77, 102, 125, 135]. Professional bodies financed one report [126] as did the Japanese government [64]. No reports received funding from the business sector. The biggest sources of funding for grey literature were Refugee charities (40%) and the Scottish government (30%) (see Fig. 2).

### Research methods and data collection

52% of reports used qualitative research methods. Forty-five reports (86%) collected data using 1–1 interviews and 24 (46%) used focus groups. Other methods of data collection included questionnaires (six studies (11%)), workshops (two studies (3.85%)) and observation (two studies (3.85%)). Oral/written evidence, guided play sessions, family case studies and participatory activity sessions were used in one report each.

28% of reports used quantitative research methods, most commonly cross section design (ten studies (36%)) and cohort design (18 studies (64%)). Information was obtained from databases including medical records, Census data and national records in 21 reports (75%). Questionnaires were used in six reports (21%). Other methods including body measurements, food diaries, blood samples, interviews and case reviews were used in 1 report each.

20% of reports used mixed methods. The most common method of data collection was questionnaires in 14 reports (70%), interviews in ten reports (50%), focus groups in seven reports (35%), workshops in three reports (13.6%), and databases in three reports (13.6%). Other methods included literature review in two reports (10%), case note reviews in two reports (10%) and one reports each used mapping and school records.

### Geographical areas of study

Ninety-one reports were situated in Scotland, of which 35 (38.5%) covered the whole country and 56 (61.5%) specified a city or area where research was undertaken. Some UK and international reports also specified the area of Scotland. The largest share of research within Scotland overall was in Glasgow with 36 reports, followed by Edinburgh with 16 reports, Lothian with six reports, Aberdeen with five reports and Grampian with three reports. The Northeast, Stirling, Highlands, Inverness, Lanarkshire, Motherwell and Selkirk had one report in each area.

There were seven international reports, three on mortality by country of birth [75, 76, 78], one on cross cultural communication [79], one on maternity care in Poland and Scotland [99], one comparing attitudes to aging in China and Scotland [137] and one on the link between birthweights and integration of migrants [64]. The remaining two reports were UK based, one on immunisation of Roma and traveller communities [117] and one on the link between ethnic diversity and mortality [104]. All the included international and UK reports documented the Scottish data separately within results.

#### Migrant population

**Fig. 3:**
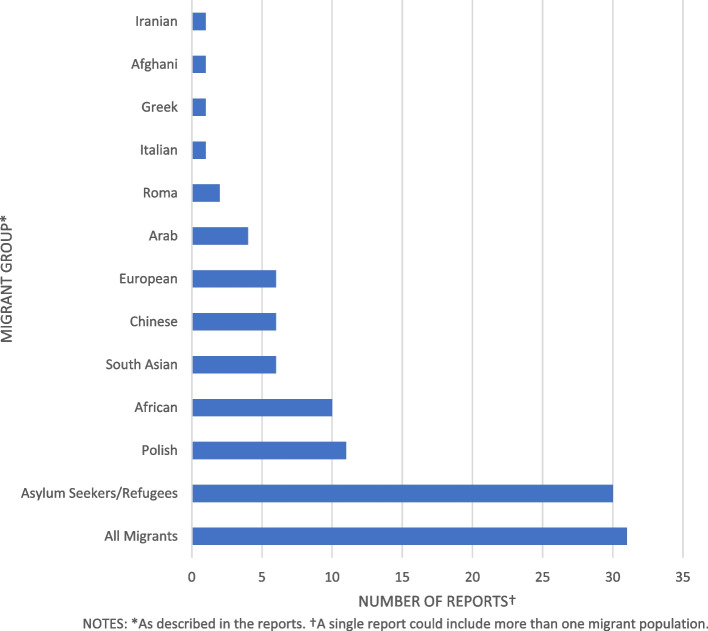
Migrant populations studied in health research in Scotland

Thirty-one reports included all migrants in the study population. The remaining reports included 30 studies on asylum seekers/refugees, 11 on Polish migrants, ten on Africans, six each on South Asians/Chinese/European, three on Arabs, and two on Roma populations (see Fig. 3). Most reports did not specify the country of origin for Asylum seekers and refugees - where country of birth was specified, reports were also included in the appropriate category.

Grey literature and peer-reviewed reports differed in population focus. The most common populations of interest in grey literature were asylum seekers/refugees consisting of 18 reports (62%) [27, 47, 54, 55, 59, 63, 70–74, 123, 125, 127, 128, 132, 134, 138] while for peer-reviewed journals 24 reports (34%) focused on all migrants [13, 35, 45, 48, 64, 76, 78–81, 104, 105, 108, 109, 113–116, 118, 120–122, 136]. 

Migrant study population also differed by local area; Glasgow city, where the majority of research occurred, had 18 reports of 36 (50%) on Asylum seekers/refugees [47, 48, 52–55, 58, 63, 70–72, 82, 83, 127, 128, 130, 138, 139] eight reports (22%) on Africans [52, 53, 84–87, 106, 107], seven reports (19%) on all migrants [45, 48, 80, 102, 104, 105, 121] and two reports (5.5%) on Roma migrants [103, 117]. Other populations had one reports each. In Edinburgh five reports of 16 (31%) were on the Polish population [56, 67, 68, 89, 90], and two reports (12.5%) on Asylum seekers/refugees [60, 133], Chinese [62, 137], South Asian [46, 119], all migrants [105, 121] and Africans [87, 107]. The remaining migrant groups had one report each. Other areas of Scotland show no clear pattern with studies in disparate migrant population groups.

**Fig. 4:**
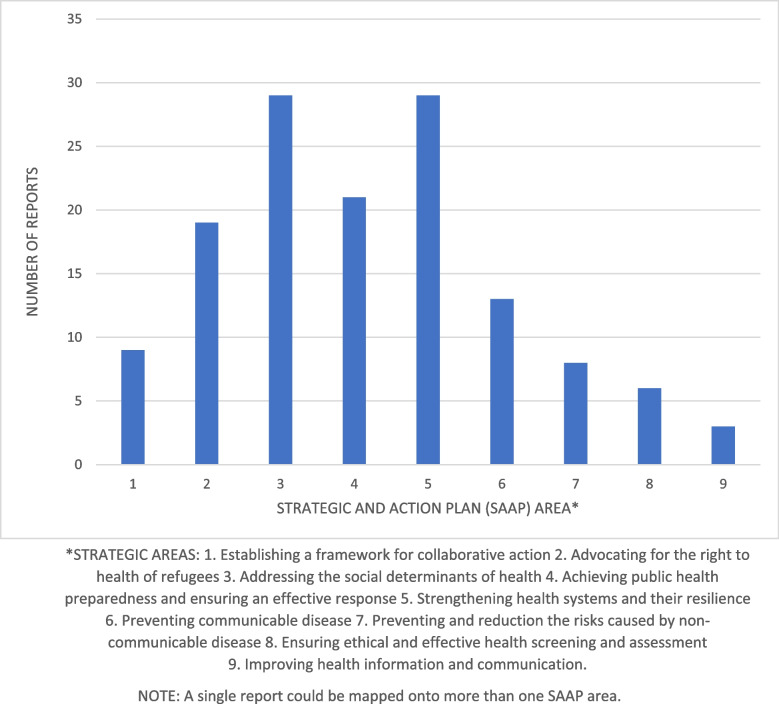
Number of reports per Strategic and Action Plan (SAAP) Area

#### SAAP Area mapping

##### 1. Establishing a framework for collaborative action

Nine reports had a primary focus on collaborative action and were categorised under SAAP area 1 (see Fig. 4) [66, 70, 72, 73, 103, 125, 129, 132, 134]. Four reports (33%) used a mixed methods study design, the remaining five reports (67%) used a qualitative design. One report [66] focused on the epidemiology of female genital mutilation and a proposed intervention strategy. One report [66] focused on the epidemiology of female genital mutilation and a proposed intervention strategy. One report [103] evaluated service provision to the Roma community in Glasgow. The remaining reports focused on refugees and asylum seekers: four [73, 125, 132, 134] evaluations of refugee integration projects, one [70] on services available to pregnant women, and one [72] an assessment of a peer-education service. One report [129] was a review of service provisions for migrants during the Covid-19 pandemic. All reports in SAAP area 1 were grey literature and three (37.5%) had a primary focus on migrant health while four (50%) focused on integration, one (11%) included data on ethnic minorities and one (11%) on services during the covid-19 pandemic. The majority (seven reports (78%)) were also categorised to another SAAP area most commonly area 2 (five studies (55%)) or area 5 (four studies (44%)).

##### 2. Advocating for the right to health of refugees

Nineteen reports focused on SAAP area 2, advocating for the right to health of refugees (see Fig. 4) [47, 52–55, 63, 70, 71, 83, 103, 123–125, 127–129, 134, 138, 140]. Sixteen reports (84%) had a qualitative study design and the remaining three (16%) reports used mixed methods. Nine reports (47%) focused on the health impact of the asylum system [52, 55, 71, 74, 123, 127–129, 138], five (26%) on health and access to care [47, 54, 83, 103, 124], two (10.5%) on maternity care [63, 70], two (10.5%) on integration services [125, 134] and one report on mental health in HIV positive migrants [53]. Nine reports (47%) had a primary focus on migrant health while the remaining 10 (53%) also involved wider social issues. The majority (15 (79%)) of reports were grey literature. All the articles in this group overlapped with another SAAP area. Area 3 is the most common joint category with ten reports (53%) followed by area 5 with seven reports (37%), area 1 shares five reports (26%), while areas 4 and 8 share one report each (5%).

##### 3. Addressing the social determinants of health

Twenty-nine reports were categorised to SAAP area 3 – addressing the social determinants of health (see Fig. 4) [13, 27, 45, 50, 52, 55, 60, 62, 63, 65, 68, 71, 74, 80–82, 91–93, 102, 112, 123, 124, 127, 128, 136–138]. The majority (14 (48%)) used a qualitative study method, eight (28%) used quantitative methodology and the remaining seven reports (24%) used mixed methods. Nineteen reports (65.5%) were peer-reviewed journals [13, 45, 50, 52, 60, 62, 63, 65, 68, 80–82, 91–93, 104, 112, 124, 136, 137] and ten (34.5%) were grey literature [27, 55, 63, 71, 74, 102, 123, 127, 128, 138]. Ten reports (34.5%) discussed the effects of the asylum system on health [27, 52, 63, 71, 74, 123, 124, 127, 128, 137] and one (3.5%) migration and health [50]. Six reports (21%) focused on culture and ethnicity [82, 92, 102, 104, 112, 137], five reports (17%) discussed economic and environmental determinants of health [13, 45, 67, 81, 93] and five reports (17%) the health impact of social activities [55, 60, 62, 80, 91]. Of the remaining reports, one [65] discussed Brexit and mental health of European migrants and one discussed the effect of coping strategies on wellbeing in Polish migrants [68]. Most reports, 18 (62%) had a primary focus on migrant health [45, 50, 52, 55, 60, 62, 63, 65, 67, 68, 71, 80–82, 91–93, 102], six reports (21%) discussed wider social factors in addition to health [74, 123, 124, 127, 128, 138]. Of the remaining reports three (10%) looked at ethnic background and country of birth [13, 112, 136], one [27] included other vulnerable groups and one [137] included people living in China and Chinese migrants to Scotland. Thirteen reports were also categorised to one or more additional SAAP area - ten (34%) were also applicable to area 2 [52, 55, 63, 71, 74, 123, 124, 127, 128, 138], three (10%) to area 5 [63, 82, 92] and one (7%) to area 4 [27].

##### 4. Achieving public health preparedness and ensuring an effective response

Twenty-one reports were assigned to SAAP area 4 (see Fig. 4) [27, 31, 35, 39, 47, 57, 64, 75–78, 94, 104, 108, 109, 111, 113, 114, 116, 120, 135] of which fourteen (67%) used quantitative research methods, four (19%) mixed methods and three (14%) qualitative methods. Thirteen (62%) reports were peer-reviewed journals [35, 59, 64, 75, 78, 104, 108, 109, 111, 113, 114, 116, 120] and eight (38%) grey literature [27, 31, 39, 47, 57, 77, 94, 135]. Most reports (12 (57%)) focused on morbidity and mortality in migrant populations [31, 35, 64, 75, 76, 78, 104, 108, 109, 113, 114, 116]. Six (29%) investigated health status and healthcare needs in migrant groups in Scotland [39, 47, 57, 77, 94, 135]. Two reports (9.5%) analysed the epidemiology of HIV infections [111, 120] and the remaining report focused on the health needs of young people during the covid-19 pandemic [27]. Nine reports (43%) had a primary focus on migrant health [39, 47, 55, 64, 75–78, 94] while eight (38%) also analysed data by ethnicity [31, 35, 104, 108, 109, 113, 114, 116]. Of the remaining reports, three (14%) included other populations within Scotland [27, 111, 120] and one (5%) included other characteristics in addition to health information [135]. Ten reports (48%) were also categorised to another SAAP area; one to area 2 [47], one to area 3 [27], four to area 5 [47, 57, 77, 135], two to area 6 [111, 120] and two to area 9 [31, 108].

##### 5. Strengthening health systems and their resilience

Twenty-nine reports were assigned to SAAP area 5 (see Fig. 4) [18, 47–49, 54, 57, 63, 69, 70, 72, 77, 79, 82, 83, 92, 95–97, 99, 101, 103, 118, 119, 126, 129, 131, 133, 135, 141] of which 23 (79%) used qualitative research methods. Three reports used quantitative methods (10.3%) and the remaining three used mixed methods (10.3%). Twelve reports (41%) examined migrants needs and experiences of health care [47, 49, 54, 57, 58, 77, 83, 95, 103, 119, 129, 135], eight (24%) focused on pregnancy and childcare [63, 70, 92, 96, 97, 99, 101, 118] and two (7%) on barriers to healthcare access [48, 131]. Two reports (7%) evaluated healthcare programmes [72, 133] and two focused on communication in primary care [79] and maternity services [69]. The remaining three reports (10%) covered sexual health [82], health information needs of Syrian refugees [126] and general practitioner training [18]. Nineteen (65.5%) were peer reviewed journals [18, 48, 49, 58, 69, 79, 82, 83, 92, 95–97, 99, 101, 118, 119, 125, 131, 133] and ten (34.5%) were grey literature [47, 54, 57, 63, 70, 72, 77, 103, 129, 135]. Twenty-one (72%) had a primary focus on migrant health [18, 47–49, 54, 57, 58, 63, 69, 70, 72, 77, 79, 82, 83, 92, 95–97, 99, 101]. Six reports (21%) included research on other characteristics or services [103, 126, 129, 131, 133, 135]. The remaining two reports (7%) included ethnic groups as well as migrants in the data [118, 119]. Nineteen reports (65.5%) were also assigned to one or more other category areas: five reports (17%) to area 1 [47, 70, 72, 103, 129], five reports (17%) to area 2 [54, 63, 83, 103, 129], three reports (10%) to area 3 [63, 82, 92], four reports (14%) to area 4 [47, 57, 77, 135], one (3.5%) to area 7 [119] and one (3.5%) to area 9 [48].

##### 6. Preventing communicable diseases

Fourteen reports were assigned to SAAP area 6 (see Fig. 4) [56, 61, 87–90, 105–107, 111, 115, 117, 120, 122] of which four (31%) used quantitative methods, five (38%) used qualitative methods and five (38%) used mixed methods. Five reports (38.5%) examined immunisation behaviour [56, 61, 89, 90, 117], five (38%) on epidemiology and treatment of HIV [106, 107, 111, 120, 122]. The remaining four reports (31%) focused on tuberculosis in healthcare workers [115], malaria [105] and sexual health services [87, 88]. Only one reports was grey literature [88], the remainder were peer-reviewed journals. Six reports (46%) had a primary focus on migrant health [56, 61, 87–90] while seven reports (54%) also included other at-risk groups in the analysis. Four reports (31%) were also assigned to another SAAP category, two (15%) to area 4 [111, 120] and two (15%) to area 8 [88, 115].

##### 7. Preventing and reducing the risks posed by non-communicable diseases

Eight reports were categorised to SAAP area 7 (see Fig. 4) [46, 51, 59, 84–86, 98, 119] of which six (75%) used qualitative research methods, one (12.5%) used quantitative methods and one (12.5%) used mixed methods. Only one report (12.5%) was grey literature [59] the remaining seven reports (87.5%) were peer-reviewed journals [48, 87, 92, 126–128, 140]. Three reports (37.5%) focused on health behaviours [51, 85, 98], two (25%) on mental health, two (25%) on diabetes and one (12.5%) on chronic disease. Seven reports(87.5%) had a primary focus on migrant health [46, 51, 59, 84–86, 98], with the remaining report (12.5%) including ethnic minority groups [119]. One report (12.5%) was also assigned to SAAP area number 5 [119].

##### 8. Ensuring ethical and effective health screening and assessment

There were six reports assigned to category 8 (see Fig. 4) [53, 88, 100, 110, 115, 121] of which two (33%) used a quantitative research method, three (50%) used a qualitative method and one used mixed methods. One report (14%) was grey literature [88] the remaining five reports (83%) were peer reviewed journals [53, 100, 110, 115, 121]. Three reports (50%) focused on cancer screening in migrant women [21, 100, 110], one (17%) analysed access to HIV testing among African migrants [53], one (17%) on T.B in healthcare workers [72] and one (17%) on sexual health [36]. Three reports (50%) had a primary focus on migrant health [53, 88, 100] while the remaining three reports (50%) included other at-risk groups in the analysis [110, 115, 121]. There were three reports which overlapped with other SAAP areas: one [53] (17%) was categorised to area 2 while two [88, 115] (33%) were categorised to area 6.

##### 9. Improving health information and communication

Three reports were assigned to SAAP area 9 (see Fig. 4) [31, 108, 130]. One of these (33%) used a qualitative approach, one (33%) used a quantitative approach and one (33%) used mixed methods. Two [108, 130] (66%) were peer-reviewed journal articles and one [31] (33%) was grey literature. Two reports (66%) focused on improving migrant demographics and health information using databases [31, 108] while one (33%) described an information-needs matrix for refugees and asylum seekers [130]. Two [31, 108] included ethnicities in the data while one [130] had a primary focus on migrant health. Two reports [31, 108] (66%) also applied to SAAP area 4 while one report [130] (33%) was in SAAP area 9 only.

## Discussion

To our knowledge this is the first scoping review conducted on migrant health in Scotland. A previous rapid literature review [94] found most research focused on health behaviours, mental health, communicable disease and use of and access to healthcare; however, the review limited migrant definition to those who had immigrated within five years and asylum seekers were not included.

In our review, the majority of reports were published from 2013 onwards, aligning with the expansion in migrant research internationally [142]. 52% used qualitative research methods, 28% used quantitative methods and 20% used mixed methods. 58% focused on migrant health: the remaining papers included other populations or health as part of a wider remit. Research funding was mostly provided by the Scottish Government, NHS, refugee charities and Universities. No studies received funding from the private sector, although this sector has the potential resource and capacity to play a key role in funding future research to improve migrant health in Scotland. Geographically, most studies took place in Glasgow (36%), nationwide (38.5%) or Edinburgh (16%) – other areas were under-represented including Aberdeen (5%), despite being the city with the largest migrant population [30]. There was a lack of studies in rural localities. These findings concur with a UK migrant health review by Burns et al. [23] where research was concentrated in larger cities and data was sparse in rural areas relative to the migrant population.

Half of the research identified that was conducted in Glasgow focused on asylum seekers/refugees. Glasgow was previously the only Scottish city to host asylum seekers [143] and currently supports the most asylum seekers of any local authority in the UK [29]. In April 2022, the UK government widened the Asylum dispersal scheme to all local authorities [144]. Around 70% of Scotland’s refugee support services are based in Glasgow and the South-west [145]. As reduced access to services may impact the health of asylum seekers, research in Glasgow may not be generalizable to other regions of Scotland.

Almost one-third (30%) of all reports focused on asylum seekers and refugees – an overrepresentation given that only 18% of migrants to the UK are asylum seekers [146] and as low as 2% of all migrants in Scotland [147]. Asylum seekers and refugees are at risk of poor health due to trauma, difficult journeys, overcrowded camps, poor nutrition and lack of access to healthcare [148]. They have worse maternity outcomes and increased rates of mental illness [149]. Increased research on health of asylum seekers and refugees is necessary due to their additional vulnerabilities [142]. However, asylum seeker’s country of origin was generally not specified. Asylum seekers have heterogenic backgrounds [150] and nationality and trauma experience affect health status [151]. Further research focused on specific nationalities of asylum seekers would enhance understanding of the health needs in this population.

Almost one-third (31%) of studies did not specify a migrant group. This concurs with a Norwegian migrant health study by Laue et al. [152] where 36% of research did not identify country of birth. Where nationality was identified, Polish, African and South Asian were most prevalent. Poles are the largest migrant group in Scotland, however for the other most common immigrant groups of Irish, Italian and Nigerian [30] there was an absence of research. No studies took place on Nigerian migrants – nine studies indicated African populations, but country of birth was not specified. Since March 2022, 23,000 Ukrainians have migrated to Scotland [153], however no studies on Ukrainians were identified currently. Research may be underway which is yet to be published.

Only one study explored the impact of Brexit on European migrants’ health despite 56% of migrants to Scotland being EU nationals [30]. Again, research may be taking place currently, which is yet to be published. No studies involved undocumented migrants despite this populations’ high rates of poor physical/mental health exacerbated by poor housing and working conditions [154]. An estimated 7.2–9.5% of the workforce in the UK are migrant workers who have higher risks of poor working conditions and injury [155]. Scotland depends on a migrant workforce for some industries such as agriculture [156] but only two research papers specified migrant workers.

Most research papers related to the right to health of refugees (SAAP 2), social determinants of health (SAAP 3), public health planning (SAAP 4) and strengthening health systems (SAAP 5). Areas with less research were frameworks for collaborative action (SAAP 1), preventing communicable disease (SAAP 6), preventing non-communicable disease (SAAP 7) and health screening and assessment (SAAP 8). Only three studies related to improving health information and communication (SAAP 9). Lebano et al. [12] conducted a literature review of migrant health in Europe and found data collection unreliable and disorganised. There is a lack of data on the numbers and types of migrants entering Scotland and research tends not to differentiate between ethnic minorities and migrants [94]. As poor-quality information hinders surveillance and planning of services SAAP area 9 is an important consideration for increased research.

Villarroel et al. [24] also found more research in SAAP areas 3 to 5 and less in areas 6 to 9. However, their study returned no results in category 1, collaborative action, or 2, the right to health of refugees, while this study assigned 9% of articles to category 1 and 19% to category 2. Most articles in our study relating to categories 1 and 2 were grey literature, which was excluded from the original Irish scoping review. This highlights a potential difference in the focus of peer-reviewed articles compared to government/refugee charity commissioned reports. Collaborative action and the right to health of refugees and asylum seekers are entwined in Scotland due to the complex policy environment; the social determinants of health such as housing, education, welfare rights and social integration are influenced by a variety of UK and Scottish statutory bodies as well as third sector organisations [157]. Despite this complexity, organisations work well together [158]. Further academic research in this area would enhance joint working practices and networks.

A scoping review in the UK [23] found similar quantities of research corresponding to SAAP areas 3, 2 and 9. However in Scotland areas 1, 5 and 8 were a combined 44% of included papers compared with 27.8% of results on health systems and structures in Burns et al’s [23] study. Almost half of the articles in SAAP areas 1,5 and 8 were grey literature, which was not included in Burns et al’s [23] review. Conversely, Burns et al. [23] found 81.9% of research in the UK related to epidemiology, equivalent to SAAP categories 4,6 and 7. In a Norwegian scoping review of migrant health [152] 65% of research was related to epidemiological data on health and disease. Only 42% of the research in this current study related to epidemiological data; the quantity of evidence was reduced by excluding combined research from the UK. As Scotland has higher mortality and morbidity than elsewhere in the UK [29] it is important to undertake further epidemiological research limited to Scotland.

### Strengths and weaknesses

Strengths of this review include the use of the WHO’s SAAP categories [7] to classify data, in accordance with the Villarroel et al’s [24] study: this means results are linked to policy on migrant health and facilitates comparability to the Irish study results. Additionally results include data on migrant groups, locality, and funding of included papers; these highlight potential omissions for future research consideration. Results include diverse research methods and published and grey literature giving a wide overview of available evidence, reported using the Preferred Reporting Items for Systematic reviews and Meta-Analyses for Scoping Reviews (PRISMA-ScR) checklist (see Additional File 3) [159]. 

Limitations included the lack of an open-access protocol and search limitations of English language and selected databases. This means some relevant reports may be omitted. Due to time and resource limitations no quality appraisal was planned for included reports. Whilst we did not synthesise the findings for each topic area and migrant group, future systematic reviews could be undertaken to address this limitation and build on this work.

## Conclusions

Immigration and ethnic diversity in Scotland have increased since 2002 which is reflected in the expansion of migrant health research. This review highlights evidence gaps including a lack of research in rural areas, undocumented migrants and migrant workers. There is a tendency to cluster asylum seekers together rather than differentiate between national groups. Within the SAAP areas there is less evidence relating to collaborative action, preventing communicable disease, preventing non-communicable disease and health screening and assessment. Further research is required on improving health information and communication for migrant populations in Scotland – a significant omission given the importance of accurate information for health service planning.

### Supplementary Information

**Supplementary Material 1.**

**Supplementary Material 2.**

**Supplementary Material 3.**

## Data Availability

All data analysed during this review comes from the papers listed in Additional file 2.

## Abbreviations

EU: European Union
HIV: Human Immunodeficiency Virus
NHS: National Health Service
SAAP: Strategy and Action Plan
SHELS: The Scottish Health and Ethnicity Linkage Study
UK: United Kingdom
WHO: World Health Organisation
