# Supplementary material for: A scoping review of academic and grey literature on migrant health research conducted in Scotland
Source: BMC Public Health. 2024 Apr 25;24:1156. doi: 10.1186/s12889-024-18628-1 (PMC11044410; doi:10.1186/s12889-024-18628-1)
Supplement: Supplementary file 1 — Supplementary Material 1. [file 12889_2024_18628_MOESM1_ESM.docx]

**Additional File 1: Database search strategies**

The following free text terms were duplicated from Villarroel et al. (2019): Asylum/Refugee/Migrant/Migrat/Emigrant/Emigrat/Immigrant/Nomad/Foreign/ Ethnic/Displaced/Stateless/State-less/Non citizen/Non-Citizen/Outsider/Newcomer/Newly Arrived/New Arrival/Recent Entrant/Non National/Non-national.

Additional relevant terms were selected from the World Health Organisation Health Network Synthesis Report 46 (Hannigan et al., 2016): Unaccompanied Minor/Unaccompanied-Minor/Human trafficking/Trafficked/Undocumented/European/Economic migrant/Economic-migrant/ Non European/Non-European/ Illegal immigrant/ illegal -immigrant.

New Scot/New-Scot was also added as this is used to refer to migrants by the devolved government and non -governmental organisations in Scotland.

The final free text terms adapted for all databases was therefore as follows:

asylum* OR refugee* OR migrant* OR migrat* OR emigrant* OR emigrat* OR immigrant* OR nomad* OR foreign* OR ethnic* OR displaced OR stateless OR state-less OR noncitizen* OR non-citizen* OR outsider OR newcomer* OR "newly arrived" OR "new arrival*" OR "recent entrant" OR "non national" OR non-national OR "unaccompanied minor" OR unaccompanied-minor OR "human trafficking" OR undocumented OR European OR "non European" OR non-European OR "economic migrant" OR economic-migrant OR "illegal immigrant" OR illegal-immigrant OR "New Scot" OR New-Scot

*CINAHL Search*

Free Text Terms Searched:

asylum* OR refugee OR migrant OR migrat* OR emigrant OR emigrat* OR immigrant OR nomad* OR foreign* OR ethnic* OR displaced OR state-less OR non-citizen OR outsider OR newcomer* OR "newly arrived" OR new-arrival OR recent-entrant OR non-national OR unaccompanied-minor OR "human trafficking" OR undocumented OR European OR non-European OR economic-migrant OR illegal-immigrant OR New-Scot OR trafficked

Index Terms Searched: Refugees OR Transients and migrants OR Immigrants OR Undocumented immigrants OR Ethnic groups OR Roma OR emigration and Immigration

Results were amalgamated and combined with AND Health AND Scotland OR Scottish. Limits were English language and published January 2002-March 2023 which identified 563 articles.

*Medline Search*

Free Text Terms searched:

asylum* OR refugee OR migrant OR migrat* OR emigrant OR emigrat* OR immigrant OR nomad* OR foreign* OR ethnic* OR displaced OR state-less OR non-citizen OR outsider OR newcomer* OR "newly arrived" OR new-arrival OR recent-entrant OR non-national OR unaccompanied-minor OR "human trafficking" OR undocumented OR European OR non-European OR economic-migrant OR illegal-immigrant OR New-Scot OR trafficked

Index Terms Searched: Transients and Migrants OR Emigrants OR Immigrants OR Human migration OR Emigration and immigration OR Undocumented immigrants OR Refugees OR Ethnic and Racial Minorities OR Human trafficking.

Results were amalgamated and combined with AND Health AND Scotland OR Scottish. Limits were English language and published January 2002-March 2023 which identified 1160 articles.

*Soc Index Search*

Free Text Terms searched:

asylum* OR refugee OR migrant OR migrat* OR emigrant OR emigrat* OR immigrant OR nomad* OR foreign* OR ethnic* OR displaced OR state-less OR non-citizen OR outsider OR newcomer* OR "newly arrived" OR new-arrival OR recent-entrant OR non-national OR unaccompanied-minor OR "human trafficking" OR undocumented OR European OR non-European OR economic-migrant OR illegal-immigrant OR New-Scot OR trafficked

Index Terms Searched: Immigrants OR Health of Immigrants OR Health of Minorities OR Medical care of immigrants OR Medical care of undocumented immigrants OR Medical care of minorities OR Medical care of refugees OR Refugees OR Health of refugees OR Foreign Workers OR Ethnic Groups OR Medical care of ethnic groups OR Newcomers OR Human trafficking OR Political refugees.

Results were amalgamated and combined with AND Health AND Scotland OR Scottish. Limits were English language and published January 2002- March 2023 which identified 196 articles.

*Web of Science Search*

Science Citation Index Expanded/Social Sciences Citation Index/ Arts and Humanities Citation Index were searched with Free Text Terms: asylum* OR refugee$ OR migrant$ OR migrat* OR emigrant$ OR emigrat* OR immigrant$ OR nomad* OR foreign* OR ethnic* OR displaced OR stateless OR state-less OR noncitizen$ OR non-citizen$ OR outsider$ OR newcomer$ OR "newly arrived" OR new-arrival$ OR recent- entrant$ OR non-national$ OR unaccompanied-minor$ OR "human trafficking" OR undocumented OR European$ OR non-European$ OR economic-migrant$ OR illegal-immigrant$ OR New-Scot$

No Index terms are available on this database. Free Text search results alone were therefore combined with AND Health AND Scotland OR Scottish. Limits were Topic field, English language and publication date January 2002- March 2023.

Grey Literature Search

The following websites were searched for relevant articles either by looking through their entire publication section or using keywords as noted below. Relevant websites were found by searching online for Scottish and UK government sites, Universities with online hubs dedicated to migrant studies, local authorities and non-governmental organisations working with migrants in the Scotland. Potentially useful reports and articles were uploaded to New RefWorks for further analysis.

*British Red Cross: searched all publications

*UK Government: searched migrant health

*Public Health Scotland: searched migrant health

*Waverley Care: searched all publications

*The Migration Observatory (University of Oxford): searched all publications in health section

*Scottish government Web: searched migrant health in research and statistics section

*Migration Scotland: searched COSLA migration, population, and diversity section using migrant health

*Scottish Refugee Council: searched all published reports

*Glasgow Refugee, Asylum, and Migration Network: searched all publications *Refugee Survival Trust: searched all publications

*Positive Action in Housing: searched all publications

*Just Right Scotland: searched all publications

+COMPAS Oxford university: searched all health publications

Google search: searched migrant health Scotland

*References*

Hannigan, A., O’Donnell, P., O’Keefe, M. and MacFarlane, A. (2016) Health evidence network synthesis report 46: How do variations in definitions of “migrant” and their application influence the access of migrants to health care services? *World Health Organisation.* Available: <https://reliefweb.int/report/world/health-evidence-network-synthesis-report-46-how-do-variations-definitions-migrant-and> (Accessed 18th January 2023).

Villarroel, N., Hannigan, A., Severoni, S., Puthoopparambil, S. and Macfarlane, A. (2019). Migrant health research in the Republic of Ireland: a scoping review. *BMC Public Health.* 19 (324). Available: <https://bmcpublichealth.biomedcentral.com/articles/10.1186/s12889-019-6651-2> (Accessed 18th January 2023).
