# Supplementary material for: A scoping review of academic and grey literature on migrant health research conducted in Scotland
Source: BMC Public Health. 2024 Apr 25;24:1156. doi: 10.1186/s12889-024-18628-1 (PMC11044410; doi:10.1186/s12889-024-18628-1)
Supplement: Supplementary file 2 — Supplementary Material 2. [file 12889_2024_18628_MOESM2_ESM.docx]

**Additional File 2: Results table**

| **Reference** | **Funding** | **Peer-reviewed journal or grey literature** | **Primary focus on migrant health** | **Migrant population** | **Primary topic** | **Geographical area** | **Study design** | **WHO SAAP area** |
| --- | --- | --- | --- | --- | --- | --- | --- | --- |
| Abdulkadir et al. (2016)^54^ | NHS Health Scotland | Grey lit. | no | Asylum seekers/refugees | Right to Health meaning | Glasgow | Qualitative | 2,5 |
| Ager et al. (2002)^60^ | Lothian Health board/Queen Margaret University | Journal | yes | Refugees | Mental health | Edinburgh | Mixed methods | 3 |
| Ahmed et al. (2010)^95^ | None declared | Journal | yes | Arab students | Primary care | Scotland | Qualitative | 5 |
| Ajetunmobi et al. (2014)^136^ | Scottish Collaboration for Public Health Research and Policy/ Glasgow Centre for Population Health. | Journal | no | undifferentiated | Breastfeeding | Scotland | Quantitative | 3 |
| Anderson et al. (2005)^51^ | Economic and Social Research Council | Journal | yes | S. Asians/Italian | Diet/ cardio-vascular disease | Glasgow | Quantitative | 7 |
| Baillot et al. (2014)^66^ | Scottish government equality fund/Rosa small grants programme | Grey lit. | yes | undifferentiated | Female Genital Mutilation | Scotland | Mixed methods | 1,5 |
| Bak-Klimek et al. (2018)^91^ | None declared | Journal | yes | Polish | Wellbeing | Scotland | Quantitative | 3 |
| Bhala et al. (2016)^114^ | University of Edinburgh | Journal | no | undifferentiated | Liver disease | Scotland | Quantitative | 4 |
| Bhopal et al. (2005)^108^ | University of Edinburgh | Journal | no | undifferentiated | Cardiovascular mortality | Scotland | Quantitative | 4,9 |
| Bhopal et al. (2012)^75^ | DG Sanco | Journal | yes | undifferentiated | Cardiovascular mortality | Denmark, England, Wales, France, Netherlands, Scotland, Sweden | Quantitative | 4 |
| Bhopal et al. (2018)^35^ | None Declared | Journal | no | undifferentiated | Mortality rates | Scotland | Quantitative | 4 |
| Bielecki et al. (2020)^89^ | None Declared | Journal | yes | Polish | Immunisation | Edinburgh | Mixed methods | 6 |
| Blake Stevenson (2007)^135^ | Glasgow City Council | Grey lit. | no | A8 Nationals | Health Service experience | Glasgow | Mixed methods | 4,5 |
| Bray et al. (2010)^96^ | None Declared | Journal | yes | Europeans | Maternity Care | Lothian | Quantitative | 5 |
| British Red Cross (2021)^74^ | The Oak Foundation | Grey lit. | no | Asylum Seekers | Destitution | Scotland | Qualitative | 2,3 |
| Cézard et al. (2022)^109^ | NHS Health Scotland, Health Protection Scotland, Chief Scientist Office | Journal | no | Undifferentiated | Ethnic health dif. | Scotland | Quantitative | 3 |
| Cheung et al. (2002)^97^ | None Declared | Journal | yes | Chinese | Maternity care | Scotland | Qualitative | 5 |
| Cheung et al. (2002)^92^ | None Declared | Journal | yes | Chinese | Childbirth | Scotland | Qualitative | 3,5 |
| Christie de-Jong et al. (2022)^110^ | None Declared | Journal | no | Arab/Asian women | Cancer Screening | Scotland | Qualitative | 8 |
| Cooper et al. (2012)^84^ | None Declared | Journal | yes | African | Chronic disease | Glasgow | Qualitative | 7 |
| Cree and Sidhva (2011)^111^ | Elton John Aids Foundation | Journal | no | African | HIV | Scotland | Mixed methods | 4,6 |
| Crowther and Lau (2019)^69^ | Iolanthe Jean Davis financial award | Journal | yes | Polish | Maternity | Scotland | Qualitative | 5 |
| Da Lomba and Murray (2014)^63^ | Comic Relief with Scottish Refugee Council, University of Strathclyde Law School | Grey lit. | yes | Asylum Seekers | Maternity | Glasgow | Qualitative | 2,3,5 |
| de Brún et al. (2015)^79^ | EU 7^th^ Framework programme | Journal | yes | Undifferentiated | Communication | Austria, England, Greece, Ireland, Scotland, Netherlands | Mixed methods | 5 |
| de Lima et al. (2007)^77^ | Communities Scotland, Aberdeen city council, Aberdeenshire council, Moray council, Scottish enterprise Grampian, NHS Grampian | Grey lit. | yes | Undifferentiated | Health Services | Grampian | Qualitative | 4,5 |
| Ezika (2014)^85^ | None Declared | Journal | yes | Africans | Smoking | Glasgow | Qualitative | 7 |
| Fassetta et al. (2016)^70^ | British Red Cross | Grey lit. | yes | Asylum Seekers | Pregnancy | Glasgow | Qualitative | 1,2,5 |
| Gallimore et al. (2021)^112^ | NHS Lothian | Journal | no | Pakistani | Contraception | Lothian | Qualitative | 3 |
| García- Medrano and Panhofer (2020)^80^ | None Declared | Journal | yes | Undifferentiated | Dance and wellbeing | Glasgow | Qualitative | 3 |
| Gillespie (2012)^123^ | Refugee Survival Trust, British Red Cross, Scottish Refugee Council | Grey lit. | no | Asylum Seekers | Destitution | Scotland | Mixed methods | 2,3 |
| Goff et al. (2021)^88^ | Scottish Government | Grey lit. | yes | Africans | Sexual health | Scotland | Mixed methods | 6,8 |
| Gorman et al. (2014)^99^ | None Declared | Journal | yes | Polish | Maternity | Scotland, Poland | Quantitative | 6 |
| Gorman and Porteous (2018)^100^ | Scottish Government Detect Cancer Early Programme | Journal | yes | Polish | Breast Screening | Lothian | Qualitative | 8 |
| Gorman et al. (2019)^90^ | None Declared | Journal | yes | Polish | Immunisation | Edinburgh | Qualitative | 6 |
| Gorman et al. (2020)^56^ | Scotland Vaccination Transformation Programme | Journal | yes | Polish | Immunisation | Edinburgh | Mixed methods | 6 |
| Gruer et al. (2018)^116^ | Chief Scientist Office, Medical Research Council, Farr institute, NHS Health Scotland, SNR Fellowship | Journal | no | Undifferentiated | Hospitalisation in ethnic groups | Scotland | Quantitative | 4 |
| Gruer et al. (2022)^113^ | Chief Scientist Office, Health Protection Scotland, NHS Health Scotland, NHS Research Scotland, The Medical Research Council, Health Data Research UK | Journal | no | Undifferentiated | Infectious disease | Scotland | Quantitative | 4 |
| Hammond (2018)^134^ | Scottish Government | Grey lit. | no | Asylum seekers and refugees | Health service | Scotland | Qualitative | 1,2 |
| Hogg et al. (2015)^101^ | Chief Scientist Office Scottish Government | Journal | yes | Pakistani/Chinese | Health visitor service | Scotland | Qualitative | 5 |
| Hopkins and Hill (2010)^124^ | None Declared | Journal | no | Asylum seekers | Health Needs | Scotland | Qualitative | 2,3 |
| Ikram et al. (2016)^78^ | None Declared | Journal | yes | Undifferentiated | Mortality Rates | Denmark, England, Wales, England, France, Netherlands, Scotland and Spain | Quantitative | 4 |
| Independent Commission of Enquiry (2022)^128^ | Not stated | Grey lit. | no | Asylum Seekers | Covid -19 | Glasgow | Qualitative | 2,3 |
| Issacs et al. (2022)^52^ | Medical Research Council | Journal | yes | African asylum seekers/refugees | Asylum system effects on health | Glasgow | Qualitative | 2,3 |
| Jackson et al. (2017)^117^ | None Declared | Journal | no | Roma | Immunisation | Glasgow, Bristol, York, London | Qualitative | 6 |
| Jamil et al. (2017)^81^ | None Declared | Journal | yes | Undifferentiated | Vitamin D/muscle strength | Aberdeen | Quantitative | 3 |
| John et al. (2021)^118^ | None Declared | Journal | no | Undifferentiated | Maternity | Scotland | Qualitative | 5 |
| Kaneoka and Spence (2019)^82^ | None Declared | Journal | yes | Asylum seekers/refugees | Sexual/ reproductive health | Glasgow | Qualitative | 3,5 |
| Karadzhov and White (2018)^86^ | None Declared | Journal | yes | African | Mental health | Glasgow | Qualitative | 7 |
| Katikireddi et al. (2004)^18^ | None Declared | Journal | yes | Asylum seekers/refugees | Primary Care | Lothian | Quantitative | 5 |
| Kearns and Whitley (2010)^102^ | Glasgow Housing Association, Scottish Government, NHS Health Scotland, NHS Greater Glasgow and Clyde | Grey lit. | yes | Undifferentiated | Wellbeing/social inclusion | Glasgow | Mixed methods | 3 |
| Kearns et al. (2017)^45^ | Scottish Government Greater Glasgow and Clyde, Glasgow Housing Association, Medical Research Council | Journal | yes | Undifferentiated | Health and deprived areas | Glasgow | Quantitative | 3 |
| Laidlaw et al. (2010)^137^ | None Declared | Journal | no | Chinese | Aging/Family support | Edinburgh and Beijing, China | Qualitative | 3 |
| Lawton et al. (2006)^119^ | Chief Scientist Office Scottish Government | Journal | yes | S. Asian | Diabetes | Edinburgh | Qualitative | 5,7 |
| Livingston et al. (2006)^120^ | Scottish Sector for Infection and Environmental Health. | Journal | no | Undifferentiated | HIV | Scotland | Quantitative | 4,6 |
| Love et al. (2007)^57^ | Scottish Health Council | Grey lit. | yes | Polish | Health Behaviours | Aberdeen | Mixed methods | 4,5 |
| Marsden et al. (2005)^138^ | Oxfam UK | Grey lit. | no | Asylum Seekers/Refugees | Destitution | Scotland | Qualitative | 2,3 |
| Marsden and Harris (2015)^125^ | European Integration Fund, British Red Cross | Grey lit. | no | Asylum Seekers | Integration/ barriers to health | Glasgow | Mixed methods | 1,2 |
| Martzoukou and Burnett (2018)^126^ | Chartered Institute of Library and Information Professionals | Journal | no | Syrian Refugees | Health Information Needs | Northeast Scotland | Qualitative | 5 |
| McCann and Mackie (2016)^94^ | None Declared | Grey lit | yes | Undifferentiated | Health Needs | Scotland | Quantitative | 4 |
| McKenna (2019)^127^ | None Declared | Grey lit. | no | Asylum Seekers | Destitution | Glasgow | Mixed methods | 2,3 |
| Nelson et al. (2021)^121^ | Chief Scientist Office Scottish Government | Journal | no | Undifferentiated | Cervical Screening | Glasgow, Edinburgh and Stirling | Quantitative | 8 |
| Noble et al. (2016)^122^ | None Declared | Journal | no | Undifferentiated | HIV | Grampian | Quantitative | 3,6 |
| O’Donnell et al. (2007)^83^ | None Declared | Journal | yes | Asylum Seekers | Access to healthcare | Glasgow | Qualitative | 2,5 |
| O’Donnell et al. (2008)^58^ | Scottish Executive Health Department | Journal | yes | Asylum Seekers | Primary healthcare | Glasgow | Qualitative | 5 |
| Oduntan and Ruthven (2019)^130^ | University of Strathclyde | Journal | no | Asylum Seekers | Information needs healthcare | Glasgow | Qualitative | 9 |
| Palattiyil and Sidhva (2015)^53^ | British Academy Grant | Journal | yes | African Asylum Seekers | HIV | Glasgow | Qualitative | 2,8 |
| Papadaki and Scott (2002)^93^ | None Declared | Journal | yes | Greek students | Diet | Glasgow | Quantitative | 3 |
| Piacentini et al. (2019)^48^ | Arts And Humanities Research Council | Journal | yes | Migrant workers and Asylum Seekers | Immigration status influence on healthcare | Glasgow | Qualitative | 5,9 |
| Pollock et al. (2017)^115^ | None Declared | Journal | no | Undifferentiated | T.B | Scotland | Quantitative | 6,8 |
| Poole and Adamson (2008)^103^ | Oxfam, Southeast Glasgow Health and Care | Grey lit. | no | Roma | Healthcare needs/access | Glasgow | Qualitative | 1,2,5 |
| Porqueddu (2017)^46^ | Chief Scientist Office Scottish Government | Journal | yes | S. Asians | Diabetes | Edinburgh | Qualitative | 7 |
| Positive Action in Housing (2021)^71^ | Positive Action in Housing | Grey lit. | Yes | Asylum Seekers | Mental Health | Glasgow | Qualitative | 2,3 |
| Quinn et al. (2011)^59^ | NHS Health Scotland | Grey lit. | yes | Asylum Seekers | Mental Health | Glasgow | Mixed methods | 7 |
| Rafnsson et al. (2013)^76^ | DG Sanco | Journal | yes | Undifferentiated | Mortality Rates | Denmark, England, France, Netherlands, Scotland, Wales, Sweden | Quantitative | 4 |
| Roshan (2005)^47^ | Scottish Executive | Grey lit. | yes | Asylum seekers/refugees | Health needs and barriers to care | Glasgow | Qualitative | 2,4,5 |
| Schofield et al. (2019)^104^ | Scotland Longitudinal Studies Centre | Journal | no | Undifferentiated | Mortality | Scotland (Glasgow), England (Manchester) and Wales | Quantitative | 2,4 |
| Scottish Government (2015)^39^ | Scottish Government | Grey lit. | no | EU/Non-EU migrants | Self-reported health | Scotland | Quantitative | 4 |
| Scottish Government (2021)^27^ | Scottish Government | Grey lit. | no | Asylum Seekers | Covid-19 experiences | Scotland | Qualitative | 3,4 |
| Sim et al. (2011)^61^ | NHS Lothian | Journal | yes | Polish | Immunisation in pregnancy | Lothian | Qualitative | 6 |
| Sime (2014)^49^ | Economic and Social Research Council | Journal | yes | Eastern European | Barriers to healthcare | Scotland | Qualitative | 5 |
| Sime and Fox (2015)^131^ | Economic and Social Research Council | Journal | no | Eastern European | Barriers to health | Scotland | Qualitative | 5 |
| Sørbye et al. (2019)^64^ | Oslo University Hospital, Norway. Ministry Of Health, Labour and Welfare, Tokyo Japan, Canada Research Chair in Applied Population Health, Authors Respective Institutions | Journal | yes | Undifferentiated | Birthweights in migrant mothers | Australia, Belgium, Canada, Denmark, Finland, Japan, Norway, Spain, Sweden, And United Kingdom (Scotland) | Quantitative | 4 |
| Spence and Zhu (2017)^98^ | None Declared | Journal | yes | Chinese | Smoking cessation | Glasgow | Qualitative | 7 |
| Steven and Munoz (2016)^50^ | The Highland Change Network | Journal | yes | EU migrants | Mental health | Highlands | Mixed methods | 3 |
| Strang (2015)^132^ | Scottish Refugee Council | Grey lit. | yes | Asylum seekers | Peer education for health/wellbeing | Glasgow | Qualitative | 1,5 |
| Strang et al. (2014)^55^ | The National Lottery | Grey lit. | no | Asylum seekers/refugees | Evaluate Holistic Integration service | Scotland | Mixed methods | 1 |
| Strang et al. (2015)^72^ | The National Lottery | Grey lit. | no | Asylum seekers/refugees | Evaluate holistic integration service | Scotland | Mixed Methods | 1 |
| Strang and Quinn (2021)^55^ | None Declared | Grey lit. | yes | Asylum seekers | Mental health | Glasgow | Qualitative | 2,3 |
| Teodorowski et al. (2021)^65^ | Robert Gordon University | Journal | yes | EU citizens | Brexit and mental health | Edinburgh, Aberdeen | Qualitative | 3 |
| Trevena et al. (2022)^129^ | Scottish Government | Grey lit. | no | Undifferentiated | Services during covid-19 | Scotland | Qualitative | 1,2,5 |
| The Scottish Ethnicity and Health Working Group (2009)^31^ | Scottish Government | Grey lit. | no | Undifferentiated | Health Status | Scotland | Mixed methods | 4,9 |
| Unger et al. (2011)^105^ | None Declared | Journal | no | Undifferentiated | Malaria | Aberdeen, Edinburgh, Inverness and Glasgow | Quantitative | 6 |
| Weir et al. (2018)^133^ | None Declared | Journal | no | Syrian refugees | Healthcare accessibility | Edinburgh | Qualitative | 5 |
| Weishaar (2008)^67^ | University of Edinburgh | Journal | yes | Polish | Stress in manual workers | Edinburgh | Qualitative | 3 |
| Weishaar (2010)^68^ | University of Edinburgh | Journal | yes | Polish | Stress | Edinburgh | Qualitative | 3 |
| Yakubu et al. (2010)^87^ | None Declared | Journal | yes | African | Sexual health | Aberdeen, Edinburgh, Dundee, Glasgow and St Andrews | Mixed methods | 6 |
| Young et al. (2014)^106^ | None Declared | Journal | no | African | HIV prevention | Glasgow, Lanarkshire, Lothian and Grampian | Qualitative | 6 |
| Young et al. (2015)^107^ | UK Medical Research Council | Journal | no | African | HIV prevention | Glasgow, Motherwell, Selkirk, Edinburgh | Qualitative | 6 |
| Zhao and Patan (2021)^62^ | University of Edinburgh | Journal | yes | Chinese students | Stress and green spaces | Edinburgh | Mixed Methods | 3 |
